# Binding and retrieval of omitted responses in complex response sequences

**DOI:** 10.3758/s13414-026-03282-z

**Published:** 2026-05-14

**Authors:** Maria Nemeth, Klaus Rothermund, Christian Frings, Birte Moeller

**Affiliations:** 1 Department of Psychology, Cognitive Psychology, Trier University, Trier, Germany; 2 Department of Psychology, Friedrich-Schiller-Universität Jena, Jena, Germany

**Keywords:** Ideomotor principle, Action planning, Actions, Binding, Retrieval

## Abstract

Human action control relies on the close interconnection of action and perception. This is possible through a binding mechanism that integrates distributed features of perceptual and action-related events within sensorimotor representations (event files). Encountering any one of these features later on can retrieve previously integrated features from memory and influence current action. Since actions are represented as their sensory consequences rather than their motor pattern, previous studies suggest that actions can be integrated into and retrieved from event representations even without being executed. However, it is still unclear whether binding and retrieval processes for omitted actions are highly automatic processes or if they can be influenced by higher-order strategies. Here we used sequential tasks to investigate whether binding and retrieval regarding omitted responses is affected by the time to prepare a response and by the likelihood of response omissions. Results indicate that binding and retrieval are highly adaptive processes that rely on the action planning of responses but operate beyond immediate action contingencies, facilitating efficient action control in future behavior.

## Introduction

Imagine you are making yourself a coffee in the morning. Your machine requires you to press four buttons in a specific sequence to create your favorite brew. But today, in the midst of your routine, you got distracted and forgot to press one of the buttons, the one for extra milk foam. Wouldn’t it be remarkable if your coffee machine nevertheless could recognize your intention, despite the omitted action, and still deliver the creamy coffee you wanted? However, while the coffee machine strongly differentiates between actual and imagined input, our cognitive system treats performed and only anticipated actions in strikingly similar ways. That is, both perceived events (i.e., perception) and movement-dependent to-be-generated events (i.e., actions) are represented by common perceptual codes.

Actions are the only way humans can influence their environment in goal-directed ways, and cognition is assumed to serve this purpose of adaptive action (e.g., Allport, 1987; Herwig, 2015). The underlying cognitive processes of intentional actions were already described in early theoretical approaches such as the *ideomotor principle* (Greenwald, 1970; James, 1890; for reviews, see Hommel, 2013; Shin et al., 2010), which assumes that actions are selected and initiated by anticipating their sensory consequences (e.g., Kunde, 2001). This requires that both perception and action are represented within shared representations (i.e., the *common code*; Hommel et al., 2001; Prinz, 1997).

Based on the ideomotor principle and earlier binding theories (Hommel et al., 2001; Logan, 1988), the Binding and Retrieval in Action Control framework (BRAC; Beste et al., 2023; Frings et al., 2020) assumes that virtually every human action is shaped by binding and retrieval processes, which integrate features of an action episode and provide adaptive shortcuts for action selection whenever these features are re-encountered. Specifically, the process of binding or integration refers to the automatic process through which temporary associations are formed between two or more feature codes within an action episode, for example between stimulus (e.g., color, shape) and response features (e.g., effector). The outcome of this process is a common episodic representation of the integrated (sensorimotor) features, referred to as the event file (Hommel, 2004b). Following this binding/integration, the process of retrieval refers to a process through which all feature codes stored within an event file are reactivated when one or more event file features reoccur in a subsequent episode (e.g., when a stimulus feature repeats; see Frings et al., 2024, for consensus definitions). Retrieval facilitates the processing of the features and supports the selection of responses consistent with the retrieved information, thereby eliminating the need for computational processes of a currently required response (Henson et al., 2014; Logan, 1988). This means that the retrieval of previously bound features (including the response) can affect current action depending on the match between the retrieved and the currently required response: If the retrieved response matches the currently required response, retrieval facilitates responding and leads to a performance benefit (i.e., lower reaction times or error rates). If a different response is required to the one retrieved, responding is impaired (i.e., increased reaction times or error rates). This pattern of performance costs and benefits due to retrieval is referred to as binding effects.

While stimulus-response bindings are considered central to most simple actions (e.g., Frings et al., 2020; Frings et al., 2007), more recent evidence suggests that bindings also provide a mechanism for linking individual actions into higher-order representations of action sequences. That is, in an ongoing action sequence bindings can also be established between individual responses within an ongoing sequence (Moeller & Frings, 2019a, 2019b). These response-response bindings are commonly studied using sequential paradigms in which dependencies between four consecutive responses are investigated within a prime-probe sequence. In the response-response binding paradigm, participants typically perform a choice reaction time task in which they execute two sequential responses in a prime phase (prime responses R1 and R2) and two sequential responses in a probe phase (probe responses R1 and R2). Crucially, it is assumed that planning and executing the prime responses leads to the binding of the two respective responses and their integration in an event file. That is, in the prime phase, the process of binding and event file integration is assumed to occur (Frings et al., 2024). Following this binding of individual responses, established response-response bindings can affect subsequent actions in the probe phase via retrieval, depending on the compatibility of the retrieved and currently required responses: If the prime R1 response is repeated as the probe R1 response (R1 response repetition), the retrieval of the associated prime R2 response can facilitate performance when the retrieved response matches the currently required probe R2 response (R2 response repetition), resulting in faster reaction times and lower error rates. Conversely, if only the probe R1 response is repeated (i.e., R1 response repetition) while the probe R2 response differs from the prime R2 response (i.e., R2 response change), retrieval of an incompatible response can impair performance due to a mismatch between the retrieved and required responses.^1^ Thus, the process of retrieval is assumed to occur in the probe, where it affects responding (Frings et al., 2024). Accordingly, response-response binding effects are only observed when both the relation of R1 (repetition vs. change) and the relation of R2 (repetition vs. change) are considered simultaneously, which statistically emerges as an interaction between the factors response-R1 relation and response-R2 relation. The occurrence of response-response binding effects is interpreted as evidence that prime responses are integrated into an event file and retrieved upon repetition of R1 in probe (Moeller & Frings, 2019b; see also Frings et al., 2024). In addition to binding effects, the literature has occasionally reported response priming effects, which are typically computed as the performance difference between response repetition and response change trials (e.g., Moeller & Frings, 2019a, 2019b). These priming effects reflect general performance advantages or disadvantages arising from feature-based retrieval processes.

Based on the ideomotor principle, it has been assumed that actions are represented as the intended perceptual feedback of a motor pattern (Stoet & Hommel, 1999), thus it follows that responses in terms of their *action plan* features are integrated into event files (Hommel et al., 2001). Evidence for this assumption comes from studies demonstrating that the mere anticipation of an action (i.e., an already formed action plan that is carried out later on) influences the planning and execution of another action (Stoet & Hommel, 1999). More recently, it was also demonstrated that responses do not need to be executed to retrieve and to be bound to other responses in a response-response binding task, but that merely intending an action – and then omitting its execution – has similar effects on binding and retrieval (Nemeth et al., 2024). Together, these findings highlight that the mere anticipation of a response is sufficient to form a feature-based the action plan, so that this response is sufficient to engage binding and retrieval mechanisms, even in the absence of a response being executed.

Importantly, an absence of action is assumed to result either from an *early decision* to omit a response or from the later inhibition of an already initiated action plan (Filevich et al., 2012; for a recent neurophysiological perspective, see Ebbesen & Brecht, 2017). Interestingly, some evidence suggests that bindings involving omitted responses occur only when a responding tendency was initially elicited. For instance, in a “planning session,” Stoet and Hommel (1999, Experiment 3) allowed participants ample time to plan an initial response (A_1_) before planning and executing a second response (B_1_, B_2_), following the typical A_1_B_1_B_2_A_2_ paradigm. In contrast, a “no-planning session” was explicitly designed to discourage advance planning of response A. That is, response planning was experimentally constrained by a very short stimulus-onset asynchrony (100 ms) between the stimuli specifying responses A and B, while the stimulus specifying response A remained visible until response B was executed. In addition, participants were explicitly instructed to plan response A only after planning and executing response B. Crucially, the authors observed response-feature overlap costs only in the planning condition, whereas the opposite pattern emerged in the no-planning condition, leading them to conclude that feature-overlap costs critically depend on intentional action planning (Stoet & Hommel, 1999). In line with this, binding of omitted responses and their contingent effects was only observed when there was an intention, that is an intention to produce the anticipated action effect, since instructed action omissions did not lead to binding of these responses and their effects (Kühn et al., 2009: Experiment 3).

Importantly for the present studies, earlier studies investigating the influence of planned but not executed actions typically presented omission cues simultaneously with the stimulus associated with the to-be-omitted response or required the eventual execution of the initially planned response at a moment later in time. However, from an ideomotor perspective, a specific prediction emerges when an omission cue *precedes* the presentation of a stimulus associated with the to-be-omitted response. That is, even though the perception or anticipation of action effects can trigger the corresponding motor action, it is reasonable that for adaptive human action control not every instance of perceiving or thinking about action effects inevitably triggers the action. Otherwise, this would result in a constant loop of action effects triggering actions, triggering action effects, and so forth (Konorski, 1967; Moeller & Pfister, 2022; Pezzulo et al., 2007). In fact, early ideomotor approaches emphasize the *desire* to achieve an action goal and thus the sensory effects of an action to be produced (Herbart, 1816, 1825; Laycock, 1860), and, that only those effects that “feel good” are capable of activating a response representation (James, 1890). In addition, when it comes to actions, “the mere presence of another idea will prevent its taking place” (p. 527; James, 1950), which could also be the idea of not to respond to a stimulus. More recent approaches explicitly emphasize the role of motivation in motor response activation. For instance, explicit strategies and expectations that participants adopt throughout an experiment are considered as influencing factors (Watson et al., 2018). The belief that an action will lead to a desired outcome also plays a crucial role in motor activation, and, importantly, goal-directed actions are assumed to be highly sensitive to affective or motivational value (Eder, 2023; Eder et al., 2015). That is, actions are only executed (and therefore should be cognitively represented) if their perceptual consequences are currently desired, thereby giving them the status of a goal (Wit & Dickinson, 2009; for action initiation that depends on action activation thresholds, see Janczyk & Kunde, 2020; Kunde et al., 2004).

Together, as action planning serves to prepare the cognitive system to produce an *intended* effect, response activation is assumed to be intentional and context dependent (Elsner & Hommel, 2001; Hommel, 2004a). Thus, the activation of response representations – and consequently, binding effects – for omitted responses should depend on additional intentions, for instance, the timing of the omission cue should influence what is planned. To the best of our knowledge, this emphasis on the context-sensitive and motivation-driven nature of anticipating to-be-expected sensory consequences of planned actions resulting in binding effects has not yet been systematically investigated.

## The present study

In the present study, we conducted two experiments to investigate the boundary conditions under which the omission of a response execution may or may not lead to binding and retrieval processes. We focused on whether an omitted response can initiate retrieval of previously bound features. Therefore, we analyzed response-response binding effects as a function of whether a critical response was executed or omitted. Crucially, the two experiments were not designed to be directly compared with each other. Instead, each experiment constitutes a targeted extension of the response-response binding effects for omitted actions reported by Nemeth et al. (2024), implementing one theoretically central modification relative to that prior work.

In both experiments, the probe R1 response served as the critical response whose execution versus omission was manipulated. The rationale of the task was that the probe R1 response constitutes the event that can initiate retrieval of the previously bound response. Accordingly, if probe R1 was omitted but response-response binding effects nevertheless emerged, this would indicate that the response was planned, and thereby able to trigger retrieval of bound features. Conversely, if omitting probe R1 eliminated response-response binding effects, this would indicate that the omitted response did not trigger retrieval, implying that this response was not intentionally planned.

Across both experiments, we used a modified response-response binding task in which participants responded to the identity of individually presented letters and digits. Each trial consisted of a sequence of four responses, within which the execution versus omission of the probe R1 response was manipulated. Salient color cues instructed participants on whether to execute or withhold the response, allowing us to dissociate response planning from response execution and to test their respective roles in initiating retrieval.

Experiment 1 examined whether response-response binding effects for omitted actions, as reported by Nemeth et al. (2024), persist when the temporal relationship between stimulus and omission cue is altered. Specifically, whereas Nemeth et al. (2024) presented the omission cue simultaneously with the stimulus, in Experiment 1 the omission cue was presented prior to the stimulus associated with the to-be-omitted response. This manipulation was theoretically motivated by ideomotor approaches emphasizing that intentional action planning depends on the anticipation of sensory consequences. Presenting the omission cue in advance was assumed to prevent intentional response planning, thereby rendering the response incapable of triggering retrieval. Relative to Nemeth et al. (2024), we therefore expected that response-response binding effects would be absent when the retrieving probe response R1 was omitted, while trials in which probe R1 was executed were expected to yield reliable binding effects.

Experiment 2 constituted a second, independent extension of Nemeth et al. (2024) and focused exclusively on the role of omission probability. While Nemeth et al. (2024) employed a 75% execution and 25% omission rate for the critical probe response, Experiment 2 reversed this proportion, using a 25% execution and 75% omission rate. All other aspects of the task, including the temporal relationship between omission cue and stimulus and the general structure of the task, were held constant relative to Nemeth et al. (2024). Our theoretical expectation was that under such conditions of frequent omission, responses could be less likely to be intentionally planned and, consequently, less likely to be bound and retrieved. This would reflect a strategic decision to avoid planning of probe response R1, because its omission was more likely than its execution. However, if action planning is a conditionally automatic process that can be influenced by current action-related goals but not by higher-order strategies, binding effects for omitted responses should be observed even if the retrieving response R1 is more frequently omitted than executed.

To foreshadow the results, relative to the binding effects reported by Nemeth et al. (2024), we found no significant response-response binding effect when the omission cue was presented prior to the stimulus that was associated with the to-be-omitted response (Experiment 1). In contrast, we found a significant response-response binding effect even when the retrieving response was omitted in 75% of all trials, making its omission the more frequent event compared to its response execution (Experiment 2).

## Experiment 1

In Experiment 1, we investigated whether presenting an omission cue before the stimulus that is associated with the to-be-omitted probe response R1 is sufficient to trigger retrieval of the bound response.

### Method

#### Participants

The sample size was calculated according to a previous study that investigated the modulation of omitting the execution of the retrieving probe response R1 on response-response binding effects, which led to a medium-sized effect *d* = 0.47 (Nemeth et al., 2024). Therefore, we planned to recruit at least *N* = 61 participants, resulting in a power of 1 – β =.95 (assuming α =.05, two-tailed; using the program G*Power 3.1.9.7; Faul et al., 2007), to obtain a significant medium-sized (*d* = 0.47) modulation of response-response binding effects. Sixty-one participants of Trier University (44 female, 17 male; 58 right-handers) with a median age of 22 years (range: 18–39 years) participated in the experiment. Participants were recruited via Trier University’s participant platform (Sona Systems; sona-systems.com) and performed the experiment online on the experimental platform Pavlovia (Peirce & MacAskill, 2018). All participants consented via an online form before participating and received course credit as compensation. This study was carried out in accordance with the ethics guidelines declared by the ethics committee of Trier University. The ethics committee of Trier University declared all simple behavioral studies in accordance with their guidelines exempt from any further examinations by the committee.

#### Design

The design comprised three within-subjects factors, namely response-R1 relation from prime to probe (response repetition vs. response change), response-R2 relation from prime to probe (response repetition vs. response change), and omission (no omission vs. probe R1 omission).

#### Apparatus and stimuli

The experiment was programmed in PsychoPy (Peirce et al., 2019;Version 2023.2.3) and ran online via Pavlovia (Peirce & MacAskill, 2018). Instructions and stimuli were shown in white (Arial font, size 35 pixels) on a gray background (RGB_255_: 128, 128, 128). Stimuli were the digits 1, 2, 3, and 4 and the letters A, B, C, and D. The outlines of different colored squares (width: 40 pixels, height: 40 pixels) were displayed in black (RGB_255_: 0, 0, 0), yellow (RGB_255_: 255, 255, 0), green (RGB_255_: 0, 128, 0), blue (RGB_255_: 0, 0, 255), or purple (RGB_255_: 128, 0, 128) around the stimuli. These squares served as cues, indicating whether participants should execute a response or refrain from doing so. Participants responded by pressing one of four keys (D, F, J or K) on the computer keyboard.

#### Procedure

Participants were tested online and were presented with instructions of the experimental procedure on the screen. Participants were instructed to position their middle and index fingers of both hands on the keys D, F, J, and K. Their task was always to press the key corresponding to each individually presented letter or number: using their left middle finger for the letter A and the number 1, their left index finger for B and 2, their right index finger for C and 3, and their left middle finger for D and 4. Participants were encouraged to respond as quickly as possible while also ensuring a high level of accuracy.

**Fig. 1:**
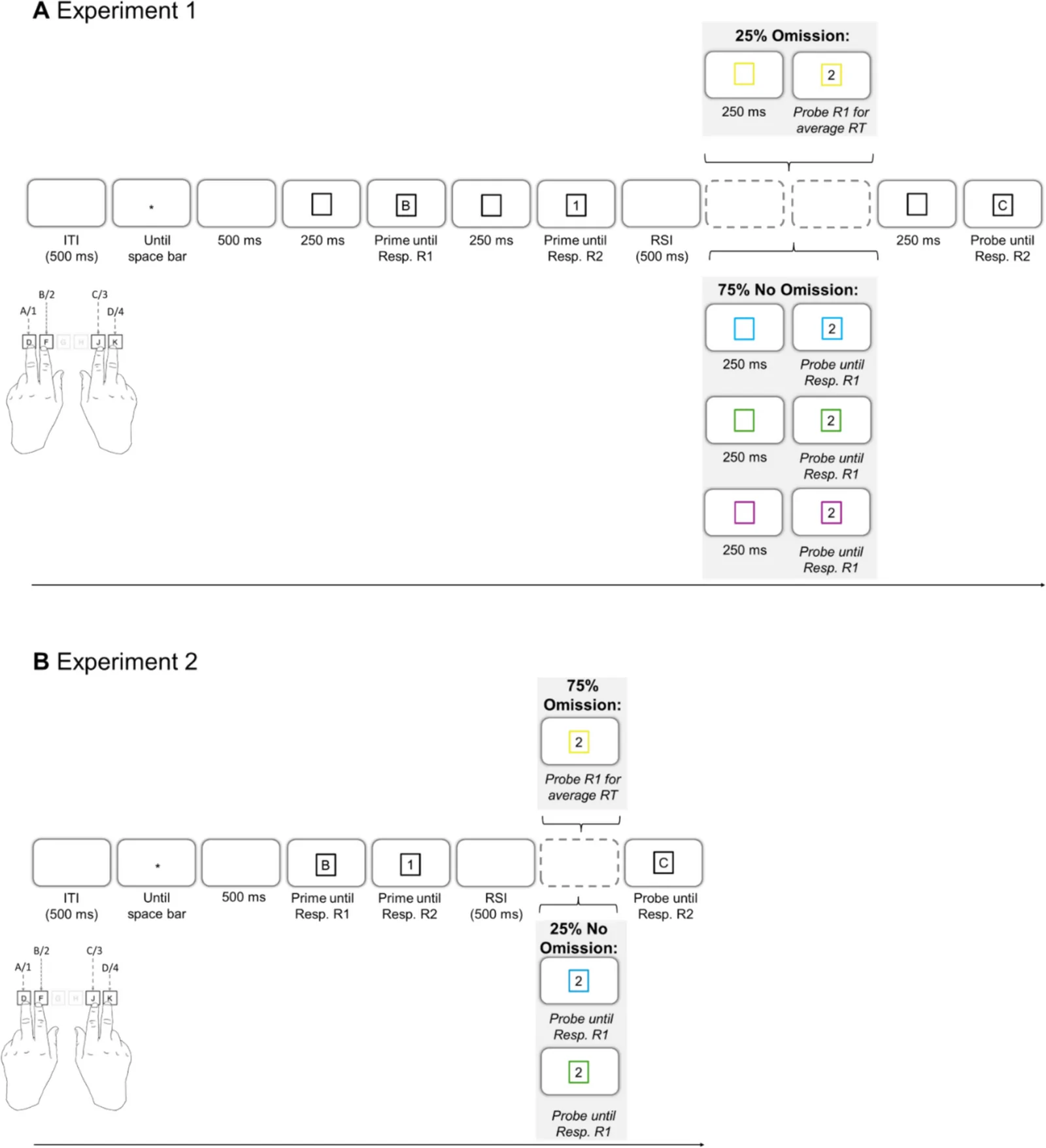
Sequence of events in one trial in Experiment 1 and Experiment 2. Participants responded with their index and middle fingers of both their hands to the identity of individually presented digits and letters. **A.** In Experiment 1, in *probe R1 omission* trials (25% of all trials), a yellow square appearing 250 ms before and subsequently surrounding the probe R1 stimulus cued participants to not execute the probe response R1. In *no omission* trials (75% of all trials; blue, green, or purple square appearing 250 ms before and subsequently surrounding the probe R1 stimulus), participants executed the probe response R1. **B.** In Experiment 2, in 75% of all trials (*probe R1 omission* trials), a yellow square cued the participants to not execute the probe response R1. In 25% of all trials (*no omission* trials) a green or blue square surrounding the probe R1 stimulus cued participants to execute the probe response R1. These are examples of a response R1 repetition and response R2 change trial. The asterisk was presented on the screen to indicate that a new trial could be started. Stimuli are not drawn to scale. RSI = response-stimulus interval, ITI = inter-trial interval

All stimuli were presented at the center of the screen. Each trial started with the presentation of an asterisk (*) at the center, signaling participants to initiate the trial by pressing the space bar, followed by a 500-ms blank space (see Fig. 1A). Then a black square was presented for 250 ms. This was followed by the first prime digit or letter, surrounded by a black square and indicating prime response R1, until the participant pressed one of the four response keys. Again, a black square was presented for 250 ms, followed by the second prime stimulus surrounded by a black square indicating prime response R2 until a response was detected. This was followed by a 500-ms blank space (response-stimulus interval; RSI). Then a colored square appeared for 250 ms; in 75% of all trials (omission condition: *no omission*) a blue, green, or purple square appeared (each 25% of the no omission trials). These cued the participants that they would have to execute the following probe response R1. In the next display, the same-colored square that was presented one display earlier surrounded the probe R1 stimulus. In 25% of the trials (omission condition: *probe R1 omission*), first a yellow square was presented for 250 ms, after that the probe R1 stimulus appeared, again surrounded by a yellow square, which cued the participants to not execute their probe response R1. In no omission trials, the presentation of the probe R1 stimulus ended upon response detection, that is, when the participant’s response was registered by the keyboard. In omission trials, the probe R1 display duration was determined individually based on the participant’s average reaction time in probe R1 no-omission trials, continuously updated across the experiment. Finally, another black square was presented for 250 ms, which was followed by the second probe stimulus always surrounded by a black square, indicating probe response R2, which remained on-screen until response detection. Between the trials, a blank screen appeared for 500 ms (inter-trial interval; ITI), before the asterisk indicated that the next trial could be started.

In 50% of the *omission* trials, participants were asked at the end of the trial to indicate which stimulus was shown in the yellow square by pressing the key on the keyboard corresponding to the identity of the stimulus (i.e., if the stimulus was A, they had to press the A on the keyboard; if the stimulus was 1, they had to press the 1 key on the keyboard). After a response was detected, the trial proceeded with the instruction to place the fingers back on the four response keys (D, F, J, K) and the trial ended by pressing the space bar. Every 48 trials, participants were prompted to take a short break.

All factors were varied orthogonally and trial-wise. In R1 repetition trials (R1r), the same response required as prime response R1 was also required as probe response R1. In R1 change trials (R1c), a different response was required as prime response R1 and probe response R1. In R2 repetition trials (R2r), the identical response required as prime response R2 was also required as probe response R2. In R2 change trials (R2c), a different response was required as prime response R2 and probe response R2. Response repetitions were always restricted to their respective positions: R1 responses could only repeat as R1, and R2 responses only as R2. Sequence of trials was randomized for each participant. For all trials, stimuli did not repeat from prime to probe. The identity of stimuli (letter vs. number) was determined randomly by the experimental program and was constrained by the response-response binding logic: For each trial, the program randomly selected a stimulus for the first prime response (R1). In response-repetition trials, the probe stimulus corresponded to the alternative stimulus mapped to the same response (e.g., “1” followed by “A”), whereas in response-change trials, the probe stimulus was randomly selected from the set of stimuli mapped to a different response, irrespective of stimulus category (letter or number). The experiment block included 480 trials (120 of each of the four conditions R1rR2r, R1rR2c, R1cR2r, R1cR2c).

Before the experimental block, participants completed a practice phase consisting of 16 trials. Participants who made more than 15% errors repeated the practice block. The practice trials were identical to the experimental trials, with the sole exception of feedback. During practice, participants received performance-contingent feedback after both prime and both probe practice displays as well as after the stimulus assessment (for a correct response: “correct”; for a wrong response: “WRONG!”; Translated from German, “richtig” and “FALSCH!”). In contrast, during the main experiment, feedback was only provided immediately following erroneous responses. Apart from this difference in feedback, the practice phase did not differ from the experimental block.

### Results

Data processing and analysis were done with R (R Core Team, 2019; R Version 4.3.0). For the analysis of reaction times (RTs), only trials with correct responses R1 and R2 in both prime and probe were included. The rate for at least one error in the prime responses (R1 or R2) was 9.0%. Probe R1 error rate in the probe R1 no omission condition was 4.4%. Probe R2 error rate was 4.2% (only including trials without errors in the previous responses). Additionally, for all analyses, trials in the probe R1 omission condition were excluded if a response was erroneously executed as probe R1 (13.4% of the remaining trials).^2^ RTs that were more than 1.5 interquartile ranges above the third quartile of each participant’s RT distribution (Tukey, 1977) and RTs below 200 ms were excluded from the analyses. Due to the exclusion of these responses, 4.1% of the trials were excluded from the RT analyses. For all analyses reported in the following, performance in probe R2 was the dependent variable of interest. If the two responses R1 and R2 in the prime were integrated, repeating prime R1 as probe R1 should trigger retrieval of the second prime response R2, thus influencing probe R2 performance. See Table 1 for mean RTs and Table 2 for error rates.

**Table 1:** Mean reaction times (in ms) for probe responses R2 in Experiment 1, as a function of R1 relation from prime to probe, R2 relation from prime to probe, and omission

	No omission		Probe R1 omission	
	R2 change	R2 repetition	R2 change	R2 repetition
R1 change	557 (71)	588 (75)	589 (90)	592 (77)
R1 repetition	579 (70)	573 (69)	581 (78)	595 (90)
R1-Priming Effect	−22 [2]	15 [3]	8 [5]	−3 [3]

*Note.* Standard deviations in parentheses. Standard error of the mean in squared brackets. R1-priming effect computed as the difference between R1 repetition and change

**Table 2:** Mean error rates (in %) for probe responses R2 in Experiment 1, as a function of R1 relation from prime to probe, R2 relation from prime to probe, and omission

	No omission		Probe R1 omission	
	R2 change	R2 repetition	R2 change	R2 repetition
R1 change	3.4 (3.0)	5.6 (4.5)	3.2 (4.2)	4.3 (5.1)
R1 repetition	4.6 (3.5)	4.7 (4.1)	3.1 (3.5)	4.6 (5.4)
R1-Priming Effect	−1.2 [0.3]	0.9 [0.3]	0.1 [0.5]	−0.4 [0.5]

*Note.* Standard deviations in parentheses. Standard error of the mean in squared brackets. R1-priming effect computed as the difference between R1 repetition and change

#### Reaction times

**Fig. 2:**
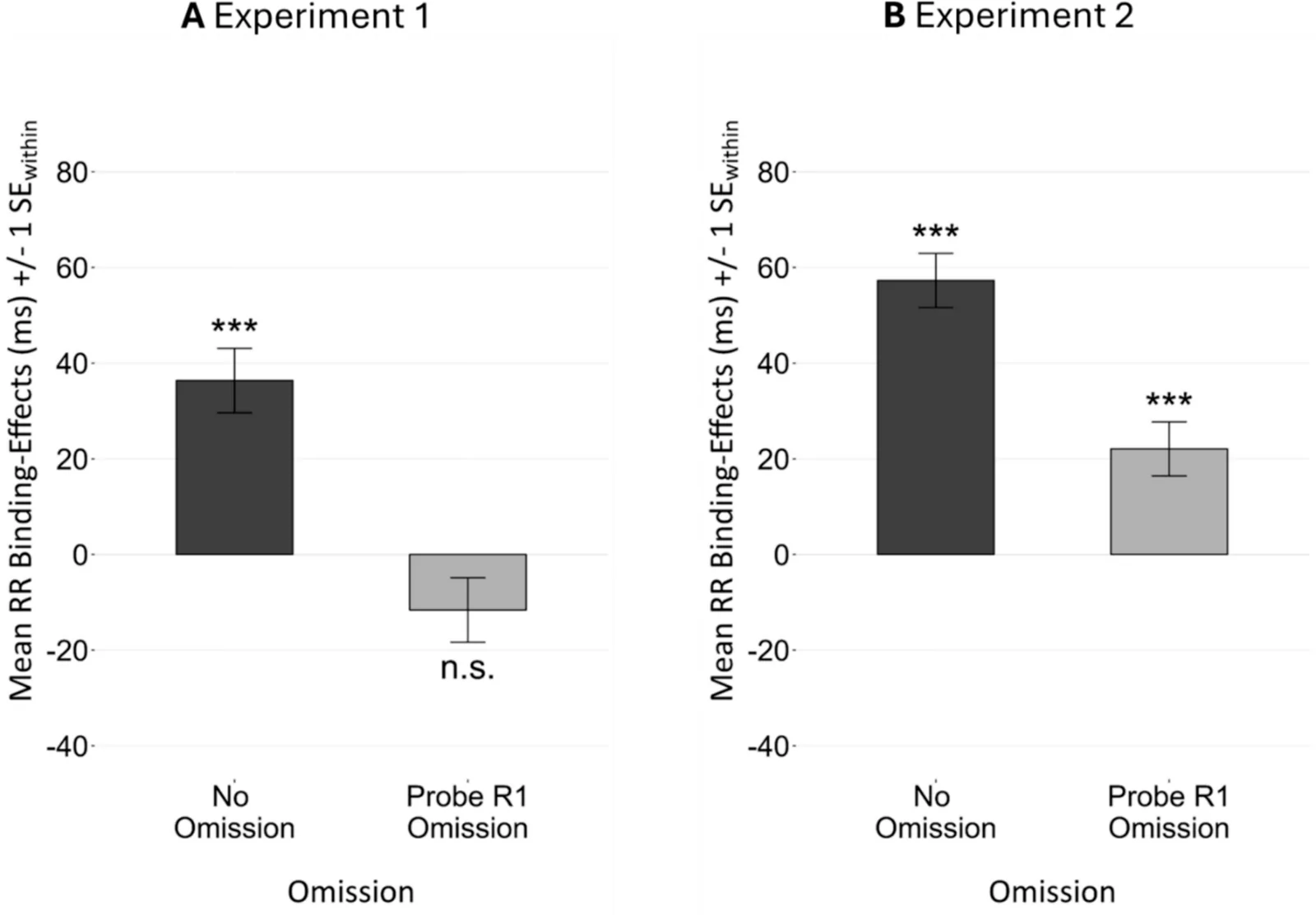
Binding effects in reaction times as a function of omission (No Omission vs. Probe R1 Omission) in Experiment 1 and Experiment 2. **A.** Response–response binding effects in reaction times as a function of omission (no omission vs. probe R1 omission) in Experiment 1. **B.** Response–response binding effects in reaction times as a function of omission (no omission vs. probe R1 omission) in Experiment 2. The response-response binding effect was computed as the advantage of probe R1 repetition over probe R1 change in probe R2 repetition trials minus the advantage of probe R1 repetition over probe R1 change in probe R2 change trials ([R1cR2r-R1rR2r]- [R1cR2c-R1rR2c]). n.s. = *p* > 0.050, *p < 0.050, **p < 0.010, ***p < 0.001

A 2 (R1 relation: repetition vs. change) × 2 (R2 relation: repetition vs. change) × 2 (omission: no omission vs. probe R1 omission) repeated-measure analysis of variance (ANOVA) on probe R2 RTs yielded a significant two-way interaction between R1 relation and R2 relation, *F*(1, 60) = 4.72, *p* =.034, ${{\eta}_{P}}^{2}$ =.07, indicating a response-response binding effect. The three-way interaction between R1 relation, R2 relation, and omission was significant, *F*(1, 60) = 25.31, *p* <.001, ${{\eta}_{P}}^{2}$ =.30, indicating larger binding effects in the no-omission condition (see Fig. 2A). Post hoc *t*-tests revealed significant response-response binding effects in the no omission condition (*M* = 36.37 ms, *SD* = 45.39 ms, *t*(60) = 6.26, *p* <.001, *d*_z_ = 0.80), but not in the probe R1 omission condition (*M* = −11.60 ms, *SD* = 68.38 ms, *t*(60) = −1.33, *p* =.190, *d*_z_ = −0.17).

For the sake of completeness, the main effect of omission was significant, *F*(1,60) = 23.22, *p* <.001, ${{\eta}_{P}}^{2}$ =.30. Participants responded faster if probe response R1 was executed (no-omission condition; *M* = 574 ms, *SD* = 72 ms) than if it was not executed (probe R1 omission condition; *M* = 589 ms, *SD* = 81 ms). Also, the main effect of R2 relation, *F*(1,60) = 8.22, *p* =.006, ${{\eta}_{P}}^{2}$ =.12, was significant. Participants responded faster if response R2 changed from prime to probe (*M* = 576 ms, *SD* = 78 ms) than if it repeated (*M* = 587 ms, *SD* = 75 ms). None of the other effects reached significance, *F*s < 3, *p*s >.09, ${{\eta}_{P}}^{2}$ s <.05.

#### Error rates

In the same analysis on probe R2 error rates, a three-way interaction between R1 relation, R2 relation, and omission was significant, *F*(1, 60) = 5.47, *p* =.023, ${{\eta}_{P}}^{2}$ =.08, indicating larger binding effects in the no omission condition. Post hoc *t*-tests revealed significant response-response binding effects in the no omission condition (*M* = 2.2%, *SD* = 2.4%, *t*(60) = 3.16, *p* =.002, *d*_z_ = 0.40), but not in the probe R1 omission condition (*M* = −0.4%, *SD* = 0.3%, *t*(60) = −0.46, *p* =.644, *d*_z_ = −0.06).

For the sake of completeness, the main effect of omission was significant, *F*(1,60) = 5.72, *p* =.020, ${{\eta}_{P}}^{2}$ =.09. Participants made more errors if probe response R1 was executed (no-omission condition; *M* = 4.6%, *SD* = 3.9%) than if it was not executed (probe R1 omission condition; *M* = 3.8%, *SD* = 4.6%). Also, the main effect of R2 relation was significant, *F*(1, 60) = 10.92, *p* =.002, ${{\eta}_{P}}^{2}$ =.15. Participants made fewer errors if response R2 changed from prime to probe (*M* = 3.6%, *SD* = 3.6%) than if it repeated (*M* = 4.8%, *SD* = 4.8%). None of the other effects reached significance, *F*s < 4, *p*s >.05, ${{\eta}_{P}}^{2}$ s <.07.

### Discussion

In Experiment 1, we found strong evidence for binding between responses when the retrieving probe response R1 was executed and thus replicated previous findings on response-response binding effects. However, no significant response-response binding effects were observed when the retrieving probe response R1 was not executed. In contrast to previous studies demonstrating response-response binding effects even when individual responses were not executed (Nemeth et al., 2024), we here presented the omission cues 250 ms *before* the stimulus that was associated with the to-be-omitted response. Our findings suggest that, for non-executed responses, an omission cue followed by merely perceiving and processing a stimulus was not sufficient to trigger the retrieval of the prime event file, thereby resulting in the absence of response-response binding effects. With that, our findings in Experiment 1 are in line with ideomotor approaches such as BRAC by highlighting the critical role of the *intention or goal* to achieve specific action consequences, which acts as a prerequisite for the actual *action planning* of responses and therefore the engagement of responses in binding and retrieval processes.

## Experiment 2

In Experiment 2, we focused on the critical role of strategies induced by the experimental context influencing binding and retrieval of responses. Specifically, we investigated a context where the omission of a critical response is the more frequent event than the execution of this response. With such an imbalance a possible strategy would be to refrain from response planning of this response by default. In this case, we would expect no response-response binding effects for omitted responses, similar to what was found in Experiment 1. Instead, a significant response-response binding effect for omitted responses would imply some form of response planning also if such planning was dispensable most of the time.

### Method

#### Participants

The power analysis in Experiment 2 was identical to Experiment 1. Sixty-one participants of Trier University (38 female, 23 male; 56 right-handers) with a median age of 22 years (range: 18–34 years) participated. Participants were recruited via Trier University’s participant platform (Sona Systems; sona-systems.com) and performed the experiment online on the experimental platform Pavlovia (Peirce & MacAskill, 2018). All participants consented via an online form before participating and received course credit as compensation. As for Experiment 1, this study was carried out in accordance with the ethics guidelines declared by the ethics committee of Trier University.

#### Design

The design was the same as in Experiment 1 and included three within-subjects factors: response R1 relation from prime to probe (response repetition vs. response change), response R2 relation from prime to probe (response repetition vs. response change), and omission (no omission vs. probe R1 omission).

#### Apparatus and stimuli

Apparatus and stimuli were the same as in Experiment 1, with the following difference: The outlines of different colored squares (width: 40 pixels, height: 40 pixels) were displayed in black (RGB_255_: 0, 0, 0), yellow (RGB_255_: 255, 255, 0), green (RGB_255_: 0, 128, 0), or blue (RGB_255_: 0, 0, 255) around the stimuli.

#### Procedure

The procedure was very similar to that in Experiment 1 with the following difference (see Fig. 1B). Importantly, all squares were exclusively presented simultaneously with the stimuli. In 25% of all trials (omission condition: *no omission*) a blue (50% of the no-omission trials) or green (50% of the no omission trials) square surrounded the probe R1 stimulus. These cued the participants to execute their probe response R1. In 75% of the trials (omission condition: *probe R1 omission*) a yellow square surrounded the probe R1 stimulus. Here, participants were cued to not execute their probe response R1.

### Results

Data processing and analysis were done with R (R Core Team, 2019; R Version 4.3.0). As in Experiment 1, performance in probe R2 was the dependent variable of interest. For the analysis of RTs, only trials with correct responses R1 and R2 in both prime and probe were included. The rate for at least one error in the prime responses (R1 or R2) was 7.2%. Probe R1 error rate in the probe R1 no omission condition was 3.4%. Probe R2 error rate was 4.4% (only including trials without errors in the previous responses). Additionally, for all analyses, trials in the probe R1 omission condition were excluded if a response was erroneously executed as probe R1 (9.6% of the remaining trials). RTs that were more than 1.5 interquartile ranges above the third quartile of each participant’s RT distribution (Tukey, 1977) and RTs below 200 ms were excluded from the analyses (4.0%). See Table 3 for mean RTs and Table 4 for error rates.

**Table 3:** Mean reaction times (in ms) for probe responses R2 in Experiment 2, as a function of R1 relation from prime to probe, R2 relation from prime to probe, and omission

	No omission		Probe R1 omission	
	R2 change	R2 repetition	R2 change	R2 repetition
R1 change	592 (88)	636 (95)	615 (88)	651 (92)
R1 repetition	622 (84)	609 (89)	633 (89)	646 (88)
R1-priming effect	−30 [3]	27 [3]	−17 [3]	5 [2]

*Note.* Standard deviations in parentheses. Standard error of the mean in squared brackets. R1-priming effect computed as the difference between R1 repetition and change

**Table 4:** Mean error rates (in %) for probe responses R2 in Experiment 2, as a function of R1 relation from prime to probe, R2 relation from prime to probe, and omission

	No omission		Probe R1 omission	
	R2 change	R2 repetition	R2 change	R2 repetition
R1 change	3.0 (4.3)	6.3 (6.3)	3.2 (2.4)	5.6 (4.1)
R1 repetition	5.3 (4.8)	3.8 (4.1)	3.8 (3.5)	4.9 (3.7)
R1-Priming Effect	−2.2 [0.5]	2.5 [0.6]	−0.6 [0.3]	0.7 [0.4]

*Note.* Standard deviations in parentheses. Standard error of the mean in squared brackets. R1-priming effect computed as the difference between R1 repetition and change

#### Reaction times

A 2 (R1 relation: repetition vs. change) × 2 (R2 relation: repetition vs. change) × 2 (prime response R2 omission: no omission vs. omission) ANOVA on probe R2 RTs yielded a significant two-way interaction between R1 relation and R2 relation, *F*(1, 60) = 77.87, *p* <.001, ${{\eta}_{P}}^{2}$ =.56, indicating a response-response binding effect. The three-way interaction between R1 relation, R2 relation, and omission was significant, *F*(1, 60) = 19.44, *p* <.001, ${{\eta}_{P}}^{2}$ =.24, indicating larger binding effects in the no omission condition (see Fig. 2B). Post hoc *t*-tests revealed significant response-response binding effects in the no omission condition (*M* = 57.29 ms, *SD* = 56.09 ms, *t*(60) = 7.98, *p* <.001, *d*_z_ = 1.02), but also in the probe R1 omission condition (*M* = 22.08 ms, *SD* = 16.64 ms, *t*(60) = 4.85, *p* <.001, *d*_z_ = 0.62).

For the sake of completeness, the main effect of omission was significant, *F*(1,60) = 23.30, *p* <.001, ${{\eta}_{P}}^{2}$ =.28. Participants responded faster if probe response R1 was executed (no-omission condition; *M* = 615 ms, *SD* = 90 ms) than if it was not executed (probe R1 omission condition; *M* = 636 ms, *SD* = 90 ms). Also, the main effect of R1 relation, *F*(1,60) = 4.04, *p* =.049, ${{\eta}_{P}}^{2}$ =.06, was significant. Participants responded faster if response R1 changed from prime to probe (*M* = 624 ms, *SD* = 93 ms) than if it repeated (*M* = 628 ms, *SD* = 88 ms). The main effect of R2 relation, *F*(1,60) = 35.07, *p* <.001, ${{\eta}_{P}}^{2}$ =.37, was also significant. Participants responded faster if response R2 changed from prime to probe (*M* = 616 ms, *SD* = 88 ms) than if it repeated (*M* = 635 ms, *SD* = 92 ms). Finally, the interaction of R2 relation and omission was significant, *F*(1,60) = 5.01, *p* =.029, ${{\eta}_{P}}^{2}$ =.08. The interaction of R1 relation and omission was not significant, *F* < 2, *p* >.1, ${{\eta}_{P}}^{2}$ s <.04.

#### Error rates

In the same analysis on error rates, the interaction between R1 relation and R2 relation was significant, *F*(1,60) = 20.98, *p* <.001, ${{\eta}_{P}}^{2}$ =.26, indicating response-response binding effects. The three-way interaction between R1 relation, R2 relation, and omission was also significant, *F*(1, 60) = 8.94, *p* =.004, ${{\eta}_{P}}^{2}$ =.13, indicating larger binding effects in the no omission condition. Post hoc analyses revealed, that the individual response-response binding effects were significantly different from zero for the no omission condition (*M* = 4.62%, *SD* = 5.10%, *t*(60) = 4.38, *p* <.001, *d*_z_ = 0.56) and also for the probe R1 omission condition (*M* = 4.37%, *SD* = 3.60%, *t*(60) = 2.22, *p* =.030, *d*_z_ = 0.28).

For the sake of completeness, the main effect of R2 relation was also significant, *F*(1, 60) = 16.28, *p* <.001, ${{\eta}_{P}}^{2}$ =.21. Participants made more errors if response R2 repeated from prime to probe (*M* = 5.2%, *SD* = 4.7%) than if it changed (*M* = 3.8%, *SD* = 3.9%). None of the other effects reached significance, *F*s < 2, *p*s >.2, ${{\eta}_{P}}^{2}$ s <.03.

### Discussion

In Experiment 2, we found evidence for binding between responses both when the retrieving response was executed and when it was omitted and thus replicated previous findings on response-response binding effects for omitted responses. Notably, we observed response-response binding effects even though the retrieving (probe R1) response was executed in only 25% of the trials. This result pattern indicates that, although the execution of the critical response was a rare event (Geyer et al., 2008), the mere presentation of a stimulus associated with the retrieving response was sufficient to trigger its cognitive representation and therefore event file retrieval. These findings suggest that an experimental context in which the execution of a specific response is less likely does not induce higher-order strategies that would prevent binding and retrieval processes from operating.

## General discussion

The literature on action control suggests the human cognitive system represents actions through their anticipated sensory consequences rather than through their motor pattern. In line with this assumption, previous studies have shown that individual responses do not have to be executed to be bound into and retrieved from cognitive representations. However, the conditions under which such binding and retrieval processes operate, particularly in relation to higher-order strategies for action planning, have remained not fully understood. The present work addressed this gap by systematically investigating how the intention to act, certainty about non-execution, and contextual action contingencies shape the binding and retrieval of responses within ongoing action sequences.

Across two experiments, we demonstrated that the current motivation to achieve an action goal modulates binding and retrieval processes when actions are omitted, whereas higher-order strategies induced by the experimental context do not prevent these processes from operating. Importantly, our data show that even intentionally omitted responses can be bound to other responses and can themselves initiate retrieval, dynamics that closely mirror those observed for executed actions. These findings characterize binding and retrieval as surprisingly adaptive, showing that even in the face of uncertainty about action execution within complex sequences, binding and retrieval remain fundamental in facilitating future action taking.

The present study investigated binding and retrieval of individual responses in relation to both the current motivation to achieve a specific action goal (Experiment 1) and higher-order motivation/strategies induced by the experimental context (Experiment 2). To do so, we employed an adapted design that has been demonstrated to reliably test for binding and retrieval of executed as well as omitted responses (Moeller & Frings, 2019b; Nemeth et al., 2024; see also, Mocke et al., 2024). In Experiment 1, we manipulated the execution (75% of all trials) versus omission of the probe response R1 (25% of all trials) – a manipulation that should target the retrieval of previously bound responses. Crucially, in contrast to previous work on response omission (Nemeth et al., 2024), we presented the omission cues 250 ms before the stimulus associated with the to-be-omitted response. This design choice was motivated by the assumption that presenting the omission cue prior to stimulus onset would prevent response planning of the associated (to-be-omitted) response. Thus, by presenting the omission at an earlier point of time than response specifying stimuli, Experiment 1 specifically tested whether binding and retrieval of responses critically depend on the intentional activation of a response goal, rather than on mere stimulus perception.

While our results clearly replicated previous findings on response-response binding effects for executed actions (e.g., Geißler et al., 2021; Moeller et al., 2025; Moeller & Frings, 2019b, 2019d, 2021), response-response binding effects did not emerge when the retrieving probe response R1 was omitted under conditions of early omission certainty. This pattern indicates that mere perception and processing of response-associated stimuli is insufficient for binding and retrieval when action planning is discouraged. Early certainty about non-execution likely prevented the intentional activation of a response goal, thereby eliminating retrieval.

In Experiment 2, we manipulated potential higher-order strategies by making the omission of the probe response R1 the more frequent event (75%) relative to execution (25%). We again observed response-response binding effects for executed actions and, importantly, demonstrated that even under conditions where execution was unlikely, retrieval processes remained operative. Crucially, omitted responses now produced significant binding effects, although these were reliably smaller than those observed for executed responses, a pattern consistent with previous findings (Nemeth et al., 2024).

One plausible explanation for reduced binding effects following omission concerns differences in feature overlap. Executed actions provide additional proximal features (e.g., tactile and proprioceptive feedback) that can serve as retrieval cues, thereby strengthening retrieval and therefore binding effects. When responses are planned but omitted, fewer shared features are available, resulting in weaker (but still reliable) retrieval. This interpretation aligns with evidence that reducing effector overlap diminishes response-response binding (Moeller & Frings, 2019d). Beyond feature overlap, comparator models of action control (e.g., Frith et al., 2000) offer an additional perspective. Discrepancies between anticipated and actual sensory consequences, particularly in cases of omission, may induce internal conflict, interference, or surprise, all of which have been associated with weaker memory encoding, reduced sense of agency, and diminished binding strength (Band et al., 2009; Hon & Yeo, 2021; Mocke et al., 2025). Thus, reduced binding for omitted actions may reflect interference between predicted action effects and experienced non-effects.

A critical theoretical contribution of the present findings concerns the nature of action representations when actions are planned but stopped. Giesen and Rothermund (2014) demonstrated that in a stimulus-response binding task, planned but ultimately stopped (prime) responses led to the retrieval of a global stop-tendency rather than the initially planned response. However, the authors themselves noted that this effect may have been driven by their use of a non-selective stop task, in which participants were instructed to stop any response upon a stop signal, without having to discriminate between specific motor responses (Giesen & Rothermund, 2014). In contrast, our findings together with those of Nemeth et al. (2024) indicate that response-response binding preserves response specificity even when actions are omitted, provided that planning has sufficiently progressed. That is, our data suggest that omitted responses are not represented as generic “no-action” events but rather retain the identity of the negated response (e.g., “not-left”), consistent with findings on nonaction–effect bindings (Kühn & Brass, 2010; Kühn et al., 2009; Weller et al., 2020).

This distinction highlights the critical role of temporal dynamics in inhibition. When inhibitory signals occur after response selection and planning have begun, specific action representations appear to be preserved and tagged with response-specific stop information (Verbruggen et al., 2005). In contrast, early inhibitory signals encountered before planning may prevent the formation of a differentiated response representation altogether, resulting in global inhibition (Aron, 2011; Giesen & Rothermund, 2014). Our Experiment 1 directly supports this distinction by showing that early omission cues abolish binding and retrieval processes.

More broadly, these findings speak to the remarkable efficiency of the cognitive system in forming event representations based on anticipatory information alone. Action planning rather than execution appears to be the critical prerequisite for establishing memory traces that guide future behavior. This conclusion challenges assumptions that localize feature integration primarily at the execution stage (e.g., Dutzi & Hommel, 2009) and substantially extends previous work that focused on action withholding with later execution (e.g., Mocke et al., 2022; Stoet & Hommel, 1999).

Importantly, goal-directed behavior requires sensitivity to the current value of action outcomes (Balleine & Dickinson, 1998). Our data suggest that the cognitive system selectively invests planning resources only when an action outcome is currently desired. Binding effects for omitted responses emerged only when omission cues coincided with response-associated stimuli, not when omission certainty preceded stimulus processing. This suggests that the mere perception of a stimulus that cues a response but crucially does not prompt the planning of that action, should not result in feature-overlap costs due to retrieval in a subsequent response – just as we found. That is, the stimulus-preceding cue in our Experiment 1 functioned as an experimental setup that discouraged participants from action planning of the retrieval triggering response. Although, as Stoet and Hommel (1999) pointed out, we also cannot be sure that this manipulation indeed targeted and prevented the planning of responses, it is clearly unnecessary to plan a response that is either specified at a later moment (as in Stoet & Hommel’s (1999) study) or not executed in the following sequence (as in our case). Taken together, Experiment 1 clearly provides empirical support for the assumption that it is the action planning of responses that leads to their integration and retrieval from event files not just perceiving and processing response-associated stimuli (see Hommel et al., 2001).

Crucially, both TEC (Hommel et al., 2001) and BRAC (Frings et al., 2020) are grounded in the ideomotor assumption that actions are represented in terms of their anticipated effects. A direct and falsifiable implication of this assumption is that if an action is planned but not executed, binding and subsequent retrieval of action features should still occur. The present study directly tested this prediction. Specifically, the absence of response-response binding effects generally following omitted probe responses would have constituted evidence against the assumption that action representations can be formed without action execution. Instead, we observed robust binding effects whenever action planning was likely to occur, even in the absence of execution, thereby providing support for this core assumption. At the same time, the elimination of binding effects when action planning was discouraged demonstrates that binding and retrieval are not inevitable consequences of mere stimulus processing, highlighting a boundary condition of automatic binding and retrieval.

Specifically, the critical role of action planning in binding and retrieval processes raises the question of whether retrieval operates as an automatic process independent of beneficial or hindering consequences for current action (e.g., Mayr & Buchner, 2010), or whether it can be influenced by higher-order strategies. Indeed, it was already demonstrated that experimental contexts influence the extent to which performance is affected by episodic retrieval processes (Frings & Wentura, 2008; Kane et al., 1997; Lowe, 1979). However, crucially, binding and retrieval effects have been observed even in contexts in which retrieval impedes efficient action regulation (Giesen & Rothermund, 2015). In such studies on stimulus-response bindings, retrieval is typically strengthened in contexts where it is beneficial (i.e., contexts involving more frequent stimulus repetitions than changes), while it is weakened if it disrupts performance more frequently (Kane et al., 1997; Lowe, 1979). Likewise, it could be argued that in Experiment 2 of the present study, action execution versus omission contingencies were manipulated, and in cases of omissions, cognitive resources were likely needed to inhibit an already formed action plan (see, Logan & Cowan, 1984). However, since response repetitions and changes were orthogonally varied, retrieval of both omitted and executed responses equally disrupted performance as much as it facilitated it. Thus, in contrast to the previously mentioned studies, our experimental context did not systematically vary the extent to which retrieval affected performance, given that omitting the retrieving response led to both benefits and costs in subsequent performance.

It is important to note that we did not explicitly ask participants whether they developed or used specific strategies due to the experimental context of response omissions. In fact, the cognitive system is tuned to recognize patterns and regularities in the environment (Garner & Felfoldy, 1970), which are assumed to implicitly guide behavior (Reber, 1989). In line with this, stimulus-response binding and retrieval processes have been found to be implicitly modulated by context-dependent contingencies, without any influence of participants’ explicit awareness of these contingencies (Giesen & Rothermund, 2015). Thus, our primary interest was not in whether participants developed specific strategies for planning their actions, but rather in whether contingencies that typically make action planning unnecessary also prevent such planning. In this regard, our approach provides a way to test the critical role of forming *intentions* in action control, particularly when uncertainty about executing actions in a sequence may lead to the development of implicit strategies on action planning. In this context, we found that binding and retrieval acted as robust and protective mechanisms that preserve the integrity of event sequences and facilitate future actions – despite interruptions or intrusions caused by action omissions.

A potential limitation of the present study concerns the constrained randomization inherent to response-response binding paradigms.^3^ Specifically, enforcing equiprobable realization of the four critical conditions necessarily alters the distribution of prime-probe relations, increasing the relative frequency of certain relations (e.g., full repetitions) and thereby introducing subtle regularities in the response sequence. Such regularities may induce a degree of predictability that can be acquired incidentally and may, in principle, contribute to the magnitude of observed binding effects. At the same time, converging evidence suggests that predictability alone is unlikely to account for the present pattern of results. Specifically, binding effects have been demonstrated under conditions without balanced frequencies (Moeller & Frings, 2019a; Experiment 3), and, in the current experiments, the fastest responses occurred in the full-change condition, which is the least predictable. Moreover, as sequence predictability was constant across our critical manipulation (omission vs. no omission), differences in individual binding effects cannot be attributed to this factor alone. More generally, response-response binding effects are more consistent with transient binding processes than with the acquisition of stable serial-order representations assumed in sequence learning accounts (see Moeller & Frings, 2019c). Nevertheless, the possibility that predictability-related influences contributed to the observed binding effects cannot be entirely ruled out, and future research may therefore benefit from employing designs that reduce structural regularities while maintaining sufficient statistical power.

In conclusion, we could demonstrate that binding and retrieval processes are reliable mechanisms that crucially depend on the current intention (Frings et al., 2020; Hommel et al., 2001; or put differently, the motivation, e.g., Wit & Dickinson, 2009) to achieve a current action goal, yet remain robust to higher-order strategies induced by the experimental context of action omissions. Taken together, retrieval appears to be a highly adaptive process that operates beyond immediate action contingencies, facilitating efficient action control in future behavior.

## Data Availability

The data of the experiments are available via the Open Science Framework (https://osf.io/n79hv/?view_only=4016fea6eb29460f90d9a8fdc842ccba). None of the experiments were preregistered.

## Footnotes

1 Note that, while response-response bindings have been shown to be independent of the original response order (i.e., the order of prime responses as repeated in the probe; Moeller & Frings, 2019c), the most common implementation of the paradigm restricts repetitions such that responses can only repeat within their respective positions, that is, prime R1 can only repeat as probe R1, and prime R2 can only repeat as probe R2.

2 Note that if only omitted trials with correct stimulus identification (loss: 11.4%) are included in the analyses, the identical pattern of results occurs.

3 We thank an anonymous reviewer for pointing this out.

## Declarations

### Open practices statement

Trial-level data for all participants across the experiments are provided on the Open Science Framework (https://osf.io/n79hv/?view_only=4016fea6eb29460f90d9a8fdc842ccba). The experiments were not preregistered.

## References

[CR1] Allport, A. (1987). Selection for action: Some behavioral and neurophysiological considerations of attention and action. In H. Heuer & A. F. Sanders (Eds.), *Perspectives on Perception and Action* (pp. 395–419). Taylor & Francis.

[CR2] Aron, A. R. (2011). From reactive to proactive and selective control: Developing a richer model for stopping inappropriate responses. *Biological Psychiatry,**69*(12), e55-68. 10.1016/j.biopsych.2010.07.024 20932513 10.1016/j.biopsych.2010.07.024 PMC3039712

[CR3] Balleine, B. W., & Dickinson, A. (1998). Goal-directed instrumental action: Contingency and incentive learning and their cortical substrates. *Neuropharmacology,**37*(4–5), 407–419. 10.1016/s0028-3908(98)00033-1 9704982 10.1016/s0028-3908(98)00033-1

[CR4] Band, G. P. H., van Steenbergen, H., Ridderinkhof, K. R., Falkenstein, M., & Hommel, B. (2009). Action-effect negativity: Irrelevant action effects are monitored like relevant feedback. *Biological Psychology,**82*(3), 211–218. 10.1016/j.biopsycho.2009.06.011 19665516 10.1016/j.biopsycho.2009.06.011

[CR5] Beste, C., Münchau, A., & Frings, C. (2023). Towards a systematization of brain oscillatory activity in actions. *Communications Biology,**6*(1), 137. 10.1038/s42003-023-04531-9 36732548 10.1038/s42003-023-04531-9 PMC9894929

[CR6] de Wit, S., & Dickinson, A. (2009). Associative theories of goal-directed behaviour: A case for animal-human translational models. *Psychologische Forschung,**73*(4), 463–476. 10.1007/s00426-009-0230-6 19350272 10.1007/s00426-009-0230-6 PMC2694930

[CR7] Dutzi, I. B., & Hommel, B. (2009). The microgenesis of action-effect binding. *Psychological Research,**73*(3), 425–435. 10.1007/s00426-008-0161-7 18810487 10.1007/s00426-008-0161-7

[CR8] Ebbesen, C. L., & Brecht, M. (2017). Motor cortex - to act or not to act? *Nature Reviews Neuroscience,**18*(11), 694–705. 10.1038/nrn.2017.119 29042690 10.1038/nrn.2017.119

[CR9] Eder, A. B. (2023). A perceptual control theory of emotional action. *Cognition & Emotion,**37*(7), 1167–1184. 10.1080/02699931.2023.2265234 37796001 10.1080/02699931.2023.2265234

[CR10] Eder, A. B., Rothermund, K., de Houwer, J., & Hommel, B. (2015). Directive and incentive functions of affective action consequences: An ideomotor approach. *Psychologische Forschung,**79*(4), 630–649. 10.1007/s00426-014-0590-4 24962237 10.1007/s00426-014-0590-4

[CR11] Elsner, B., & Hommel, B. (2001). Effect anticipation and action control. *Journal of Experimental Psychology: Human Perception and Performance,**27*(1), 229–240. 10.1037/0096-1523.27.1.229 11248937 10.1037//0096-1523.27.1.229

[CR12] Faul, F., Erdfelder, E., Lang, A.-G., & Buchner, A. (2007). G*power 3: A flexible statistical power analysis program for the social, behavioral, and biomedical sciences. *Behavior Research Methods,**39*(2), 175–191. 10.3758/bf03193146 17695343 10.3758/bf03193146

[CR13] Filevich, E., Kühn, S., & Haggard, P. (2012). Intentional inhibition in human action: The power of “no.” *Neuroscience and Biobehavioral Reviews,**36*(4), 1107–1118. 10.1016/j.neubiorev.2012.01.006 22305996 10.1016/j.neubiorev.2012.01.006

[CR14] Frings, C., Beste, C., Benini, E., Möller, M., Dignath, D., Giesen, C. G., Hommel, B., Kiesel, A., Koch, I., Kunde, W., Mayr, S., Mocke, V., Moeller, B., Münchau, A., Parmar, J., Pastötter, B., Pfister, R., Philipp, A. M., Qiu, R., … Schmalbrock, P. (2024). Consensus definitions of perception-action-integration in action control. *Communications Psychology,**2*(1), 1–5. 10.1038/s44271-023-00050-9 39242844 10.1038/s44271-023-00050-9 PMC11332160

[CR15] Frings, C., Hommel, B., Koch, I., Rothermund, K., Dignath, D., Giesen, C., Kiesel, A., Kunde, W., Mayr, S., Moeller, B., Möller, M., Pfister, R., & Philipp, A. (2020). Binding and Retrieval in Action Control (BRAC). *Trends in Cognitive Sciences,**24*(5), 375–387. 10.1016/j.tics.2020.02.004 32298623 10.1016/j.tics.2020.02.004

[CR16] Frings, C., Rothermund, K., & Wentura, D. (2007). Distractor repetitions retrieve previous responses to targets. *Quarterly Journal of Experimental Psychology,**60*(10), 1367–1377. 10.1080/17470210600955645 10.1080/17470210600955645 17853245

[CR17] Frings, C., & Wentura, D. (2008). Separating context and trial-by-trial effects in the negative priming paradigm. *European Journal of Cognitive Psychology,**20*(2), 195–210. 10.1080/17470910701363090

[CR18] Frith, C. D., Blakemore, S. J., & Wolpert, D. M. (2000). Abnormalities in the awareness and control of action. *Philosophical Transactions of the Royal Society of London. Series B: Biological Sciences,**355*(1404), 1771–1788. 10.1098/rstb.2000.0734 11205340 10.1098/rstb.2000.0734 PMC1692910

[CR19] Garner, W., & Felfoldy, G. L. (1970). Integrality of stimulus dimensions in various types of information processing. *Cognitive Psychology,**1*(3), 225–241. 10.1016/0010-0285(70)90016-2

[CR20] Geißler, C. F., Frings, C., & Moeller, B. (2021). Illuminating the prefrontal neural correlates of action sequence disassembling in response-response binding. *Scientific Reports,**11*(1), Article 22856. 10.1038/s41598-021-02247-6 34819541 10.1038/s41598-021-02247-6 PMC8613220

[CR21] Geyer, T., Müller, H. J., & Krummenacher, J. (2008). Expectancies modulate attentional capture by salient color singletons. *Vision Research,**48*(11), 1315–1326. 10.1016/j.visres.2008.02.006 18407311 10.1016/j.visres.2008.02.006

[CR22] Giesen, C. G., & Rothermund, K. (2014). You better stop! Binding “stop” tags to irrelevant stimulus features. *Quarterly Journal of Experimental Psychology,**67*(4), 809–832. 10.1080/17470218.2013.834372 10.1080/17470218.2013.834372 24131363

[CR23] Giesen, C. G., & Rothermund, K. (2015). Adapting to stimulus-response contingencies without noticing them. *Journal of Experimental Psychology: Human Perception and Performance,**41*(6), 1475–1481. 10.1037/xhp0000122 26389614 10.1037/xhp0000122

[CR24] Greenwald, A. G. (1970). Sensory feedback mechanisms in performance control: With special reference to the ideo-motor mechanism. *Psychological Review,**77*(2), 73–99. 10.1037/h0028689 5454129 10.1037/h0028689

[CR25] Henson, R. N., Eckstein, D., Waszak, F., Frings, C., & Horner, A. J. (2014). Stimulus-response bindings in priming. *Trends in Cognitive Sciences,**18*(7), 376–384. 10.1016/j.tics.2014.03.004 24768034 10.1016/j.tics.2014.03.004 PMC4074350

[CR26] Herbart, J. F. (1816). *Lehrbuch der Psychologie*. Unzer.

[CR27] Herbart, J. F. (1825). *Psychologie als Wissenschaft neu gegründet auf Erfahrung, Metaphysik und Mathematik*. *2*. Unzer.

[CR28] Herwig, A. (2015). Linking perception and action by structure or process? Toward an integrative perspective. *Neuroscience and Biobehavioral Reviews,**52*, 105–116. 10.1016/j.neubiorev.2015.02.013 25732773 10.1016/j.neubiorev.2015.02.013

[CR29] Hommel, B. (2004). Coloring an action: Intending to produce color events eliminates the Stroop effect. *Psychologische Forschung,**68*(2–3), 74–90. 10.1007/s00426-003-0146-5 14634808 10.1007/s00426-003-0146-5

[CR30] Hommel, B. (2004). Event files: Feature binding in and across perception and action. *Trends in Cognitive Sciences,**8*(11), 494–500. 10.1016/j.tics.2004.08.007 15491903 10.1016/j.tics.2004.08.007

[CR31] Hommel, B. (2013). Ideomotor Action Control: On the Perceptual Grounding of Voluntary Actions and Agents. In W. Prinz, M. Beisert, & A. Herwig (Eds.), *Action Science* (pp. 112–136). MIT Press. 10.7551/mitpress/9780262018555.003.0005

[CR32] Hommel, B., Müsseler, J., Aschersleben, G., & Prinz, W. (2001). The theory of event coding (TEC): A framework for perception and action planning. *Behavioral and Brain Sciences,**24*(5), 849–878. 10.1017/s0140525x01000103 12239891 10.1017/s0140525x01000103

[CR33] Hon, N., & Yeo, N. (2021). Having a sense of agency can improve memory. *Psychonomic Bulletin & Review,**28*(3), 946–952. 10.3758/s13423-020-01849-x 33415660 10.3758/s13423-020-01849-x

[CR34] James, W. (1890). *The principles of psychology, Vol. 2*. Harvard University Press. 10.1037/10538-000

[CR35] James, W. (1950). *The principles of psychology: Vol. 2.* (Original work published 1890). New York, NY: Dover.

[CR36] Janczyk, M., & Kunde, W. (2020). Dual tasking from a goal perspective. *Psychological Review,**127*(6), 1079–1096. 10.1037/rev0000222 32538637 10.1037/rev0000222

[CR37] Kane, M. J., May, C. P., Hasher, L., Rahhal, T., & Stoltzfus, E. R. (1997). Dual mechanisms of negative priming. *Journal of Experimental Psychology: Human Perception and Performance,**23*, 632–650. 9180038 10.1037//0096-1523.23.3.632

[CR38] Konorski, J. (1967). *Integrative activity of the brain*. University of Chicago Press. https://psycnet.apa.org/record/1967-35012-000

[CR39] Kühn, S., Elsner, B., Prinz, W., & Brass, M. (2009). Busy doing nothing: Evidence for nonaction–effect binding. *Psychonomic Bulletin & Review,**16*(3), 542–549. 10.3758/PBR.16.3.542 19451382 10.3758/PBR.16.3.542

[CR40] Kunde, W. (2001). Response-effect compatibility in manual choice reaction tasks. *Journal of Experimental Psychology: Human Perception and Performance,**27*(2), 387–394. 10.1037/0096-1523.27.2.387 11318054 10.1037//0096-1523.27.2.387

[CR41] Kunde, W., Koch, I., & Hoffmann, J. (2004). Anticipated action effects affect the selection, initiation, and execution of actions. *The Quarterly Journal of Experimental Psychology Section a,**57*(1), 87–106. 10.1080/02724980343000143 10.1080/02724980343000143 14681005

[CR42] Laycock, T. (1860). Mind and Brain: Or, the Correlations of Consciousness and Organisation; with Their Applications to Philosophy, Zoology, Physiology, Mental Patholgy, and the Practice of Medicine. *Sutherland & Knox*. 10.1037/12161-000 PMC5178519 30163413

[CR43] Logan, G. D. (1988). Toward an instance theory of automatization. *Psychological Review,**95*(4), 492–527. 10.1037/0033-295X.95.4.492

[CR44] Logan, G. D., & Cowan, W. B. (1984). On the ability to inhibit thought and action: A theory of an act of control. *Psychological Review,**91*(3), 295–327. 10.1037/0033-295x.91.3.295 10.1037/a0035230 24490789

[CR45] Lowe, D. G. (1979). Strategies, context, and the mechanism of response inhibition. *Memory & Cognition,**7*(5), 382–389. 10.3758/BF03196943

[CR46] Mayr, S., & Buchner, A. (2010). Episodic retrieval processes take place automatically in auditory negative priming. *European Journal of Cognitive Psychology,**22*(8), 1192–1221. 10.1080/09541440903409808

[CR47] Mocke, V., Beste, C., Pastötter, B., & Kunde, W. (2024). Action plan discarding leads to unbinding of action features. *Journal of Experimental Psychology: Human Perception and Performance,**50*(9), 903–917. 10.1037/xhp0001219 39052421 10.1037/xhp0001219

[CR48] Mocke, V., Holzmann, P., Hommel, B., & Kunde, W. (2022). Beyond left and right: Binding and retrieval of spatial and temporal features of planned actions. *Journal of Cognition,**5*(1), Article 6. 10.5334/joc.197 36072095 10.5334/joc.197 PMC9400704

[CR49] Mocke, V., Kunde, W., & Schreiner, M. R. (2025). *Compatible Effects Enhance Short-Term Action-Effect Binding*. Center for Open Science. 10.31219/osf.io/2g36z_v2 10.1037/xlm0001503 40608465

[CR50] Moeller, B., Beste, C., Münchau, A., & Frings, C. (2025). Large scale event segmentation affects the microlevel action control processes. *Journal of Experimental Psychology: General,**154*(4), 969–979. 10.1037/xge0001681 39804386 10.1037/xge0001681

[CR51] Moeller, B., & Frings, C. (2019). Binding processes in the control of nonroutine action sequences. *Journal of Experimental Psychology: Human Perception and Performance,**45*(9), 1135–1145. 10.1037/xhp0000665 31144858 10.1037/xhp0000665

[CR52] Moeller, B., & Frings, C. (2019). From simple to complex actions: Response-response bindings as a new approach to action sequences. *Journal of Experimental Psychology. General,**148*(1), 174–183. 10.1037/xge0000483 30211579 10.1037/xge0000483

[CR53] Moeller, B., & Frings, C. (2019). Lost time: Bindings do not represent temporal order information. *Psychonomic Bulletin & Review,**26*(1), 325–331. 10.3758/s13423-018-1493-y 29869024 10.3758/s13423-018-1493-y

[CR54] Moeller, B., & Frings, C. (2019). Response-response binding across effector-set switches. *Psychonomic Bulletin & Review,**26*(6), 1974–1979. 10.3758/s13423-019-01669-8 31654376 10.3758/s13423-019-01669-8

[CR55] Moeller, B., & Frings, C. (2021). Remote binding counts: Measuring distractor-response binding effects online. *Psychological Research,**85*(6), 2249–2255. 10.1007/s00426-020-01413-1 32894340 10.1007/s00426-020-01413-1 PMC8357652

[CR56] Moeller, B., & Pfister, R. (2022). Ideomotor learning: Time to generalize a longstanding principle. *Neuroscience and Biobehavioral Reviews,**140*, Article 104782. 10.1016/j.neubiorev.2022.104782 35878792 10.1016/j.neubiorev.2022.104782

[CR57] Nemeth, M., Frings, C., Schmalbrock, P., & Moeller, B. (2024). No need to execute: Omitted responses still yield response-response binding effects. *Journal of Experimental Psychology: Human Perception and Performance,**50*(12), 1196–1205. 10.1037/xhp0001251 39388103 10.1037/xhp0001251

[CR58] Peirce, J., Gray, J. R., Simpson, S., MacAskill, M., Höchenberger, R., Sogo, H., Kastman, E., & Lindeløv, J. K. (2019). Psychopy2: Experiments in behavior made easy. *Behavior Research Methods,**51*(1), 195–203. 10.3758/s13428-018-01193-y 30734206 10.3758/s13428-018-01193-y PMC6420413

[CR59] Peirce, J., & MacAskill, M. (2018). *Building experiments in PsychoPy*. SAGE Publications.

[CR60] Pezzulo, G., Baldassarre, G., Butz, M. V., Castelfranchi, C., & Hoffmann, J. (2007). From Actions to Goals and Vice-Versa: Theoretical Analysis and Models of the Ideomotor Principle and TOTE. In (pp. 73–93). Springer, Berlin, Heidelberg. 10.1007/978-3-540-74262-3_5

[CR61] Prinz, W. (1997). Perception and action planning. *European Journal of Cognitive Psychology,**9*(2), 129–154. 10.1080/713752551

[CR62] R Core Team. (2019). *A language and environment for statistical computing.* R Foundation for Statistical Computing. https://www.R-project.org/

[CR63] Reber, A. S. (1989). Implicit learning and tacit knowledge. *Journal of Experimental Psychology: General,**118*(3), 219–235. 10.1037/0096-3445.118.3.219

[CR64] Shin, Y. K., Proctor, R. W., & Capaldi, E. J. (2010). A review of contemporary ideomotor theory. *Psychological Bulletin,**136*(6), 943–974. 10.1037/a0020541 20822210 10.1037/a0020541

[CR65] Stoet, G., & Hommel, B. (1999). Action planning and the temporal binding of response codes. *Journal of Experimental Psychology: Human Perception and Performance,**25*(6), 1625–1640. 10.1037/0096-1523.25.6.1625

[CR66] Tukey, J. W. (1977). *Exploratory data analysis*. *Addison-Wesley series in behavioral science Quantitative methods*. Addison-Wesley.

[CR67] Verbruggen, F., Liefooghe, B., & Vandierendonck, A. (2005). On the difference between response inhibition and negative priming: Evidence from simple and selective stopping. *Psychological Research,**69*(4), 262–271. 10.1007/s00426-004-0177-6 15750869 10.1007/s00426-004-0177-6

[CR68] Watson, P., Wiers, R. W., Hommel, B., & de Wit, S. (2018). Motivational sensitivity of outcome-response priming: Experimental research and theoretical models. *Psychonomic Bulletin & Review,**25*(6), 2069–2082. 10.3758/s13423-018-1449-2 29468416 10.3758/s13423-018-1449-2 PMC6267533

[CR69] Weller, L., Schwarz, K. A., Kunde, W., & Pfister, R. (2020). Something from nothing: Agency for deliberate nonactions. *Cognition,**196*, 104136. 10.1016/j.cognition.2019.104136 10.1016/j.cognition.2019.104136 31760322

